# Preference for Male Facial Masculinity as a Function of Mental Rotation Ability in Gay and Bisexual Men, but Not in Heterosexual Men and Women in China

**DOI:** 10.3389/fpsyg.2019.02419

**Published:** 2019-10-25

**Authors:** Lijun Zheng

**Affiliations:** Faculty of Psychology, Southwest University, Chongqing, China

**Keywords:** facial masculinity preference, mental rotation, homogamy, gay and bisexual men, partner choice

## Abstract

This study examined the association between mental rotation ability and facial masculinity preference in gay and bisexual men in China. The participants (436 gay/bisexual men, 132 heterosexual men, and 254 heterosexual women) completed an online Shepard and Metzler-type mental rotation task and a forced-choice preference task of 10 pairs of masculinized/feminized male faces. The results revealed that mental rotation ability was significantly associated with preference for masculinized faces in both gay and bisexual men. There were no significant correlations between mental rotation ability and facial masculinity preference in both heterosexual men and women. The findings imply homogamy in partner preference in gay and bisexual men in terms of masculinity.

## Introduction

Masculine male facial characteristics (e.g., a pronounced brow and large jaw) are positively related to the circulating testosterone levels (Roney et al., 2006) and they may be regarded as a cue to good health (Gangestad and Simpson, 2000; Rhodes et al., 2003; Thornhill and Gangestad, 2006). Various factors contribute to individual differences in male facial masculinity preference in women, including relationship status (Sacco et al., 2012), self-reported attractiveness (Little and Mannion, 2006), sexual desire (Jones et al., 2011), sociosexuality (Glassenberg et al., 2010; Stower et al., 2019), and menstrual cycle (Penton-Voak et al., 1999; Johnston et al., 2001).

In general, gay and bisexual men prefer masculinized faces over feminized faces (Glassenberg et al., 2010; Zheng et al., 2013; Zheng and Zheng, 2015). However, some studies found no overriding preference among homosexual males for either masculine or feminine facial features (Valentová et al., 2013; Welling et al., 2013). There are also extensive individual differences in facial masculinity preference in gay and bisexual men for some variables, including sex role identity (i.e., tops, versatiles, and bottoms; Zheng et al., 2013), relationship status (Zheng, 2019), sexism (Zheng and Zheng, 2015), and pathogen disgust (Zheng et al., 2016).

Previous empirical evidence indicates homogamy in partner preference in gay men. Gay men prefer potential partners who are similar to themselves in personality (Štěrbová et al., 2017), height (Valentova et al., 2014, 2016), and beardedness (Valentova et al., 2017). Gay men prefer masculine men, and preference for masculinity may be related to the participants’ own levels of masculinity (Bailey et al., 1997). Gay men with positive attitudes toward masculine gay men preferred masculine male faces, voices, bodies, and personality traits (Zheng and Zheng, 2016). Moreover, homogamy in masculinity-femininity is positively linked to relationship quality in gay male couples (Bártová et al., 2017). Overall, previous studies indicated that gay men’s self-perceived masculinity and masculinity norms were related to masculinity preference in partner choice.

It is possible that gay men’s masculinity in the cognitive domain would be related to facial masculinity preference, based on the theory of homogamy. Mental rotation ability has been found to be related to masculine traits, such as systemizing (Cook and Saucier, 2010; Zheng and Zheng, 2017). In general, men significantly outperform women on tests of mental rotation ability (e.g., Voyer et al., 1995; Peters et al., 2007). Therefore, mental rotation ability is a masculine cognitive capacity and may be related to facial masculinity preference in gay men.

Mental rotation ability has been found to be highly related to face processing (e.g., Lewis, 2001; Stevenage and Osborne, 2006). The majority of previous studies manipulated the degree of facial masculinization and feminization based on the same original face (Rowland and Perrett, 1995; Penton-Voak et al., 1999). There were only minor differences between masculinized and feminized faces. Face processing-related ability might be helpful for individuals to determine their preferred facial features; if one could not detect the tiny differences between masculinized and feminized faces, on the other hand, this process would be difficult. If mental rotation ability is related to male facial masculinity preference *via* face processing ability, this effect would be true for both gay men and heterosexual women. Thus, I also assessed the relationship between mental rotation and preference for male facial masculinity in heterosexual women to test this hypothesis.

In addition, previous studies indicated that intrasexually competitive individuals similarly indicate heightened preferences toward good gene cues in same-sex faces (e.g., Sacco et al., 2009; Brown and Sacco, 2017). Gay men could view masculine men as competition as much as prospective mates. Therefore, it is possible that gay men with high mental rotation ability would prefer masculine faces due to the potential competition posed by masculine faces. To test this hypothesis, I also assessed the relationship between mental rotation and preference for male facial masculinity in heterosexual men. If competition contributes to facial masculinity preference, both gay and heterosexual men would show a similar pattern.

Overall, this study examined the association between mental rotation ability and facial masculinity preference in gay/bisexual men and heterosexuals in China. I hypothesized that gay and bisexual men with high mental rotation ability would prefer more masculine faces, based on the theory of homogamy. I also examined this association in heterosexual women to test the potential explanation of face processing ability. In addition, this association was also examined in heterosexual men to test whether this association was based on intrasexual competition rather than homogamy.

## Methods

### Participants

The required sample size was 138 participants, as indicated by GPower3.1, targeting power of 0.95 in a tow-tailed correlation. The participants in this study were 327 self-identified gay men and 109 self-identified bisexual men aged between 16 and 50 years (*M* = 23.4, SD = 5.6). The control sample included 132 heterosexual men and 245 heterosexual women aged between 17 and 49 years (*M* = 27.1, SD = 5.9). Of these, 35 (4.3%) had a junior high school education or lower, 136 (16.7%) had a senior high school education, 576 (70.8%) had a college education, and 66 (8.1%) had a postgraduate education or higher. In terms of employment, 513 participants (63.1%) were employed full-time and 300 (36.9%) were students.

### Procedure

The study was conducted online *via* a Chinese survey website^1^. The written informed consent was inferred by completion of the online study. Gay and bisexual participants were recruited *via* several Chinese websites that cater to gay people (including forums and QQ groups: a popular chat software in China) in February 2019. Heterosexual participants were recruited *via* Weidiaocha, a professional survey platform, in August 2019. They first responded to questions about their demographic information, including sex (male or female), age, sexual orientation (homosexual, bisexual, or heterosexual), occupation, and education. Then, they completed the facial masculinity preference measures. Finally, the participants performed a mental rotation task. The three sections were presented on three webpages. The participants could not enter the next section until they had finished all of the items in the previous section. If they missed any item, the system would remind them and locate the missing items.

### Measures

#### Facial Masculinity Preference

**Figure 1:**
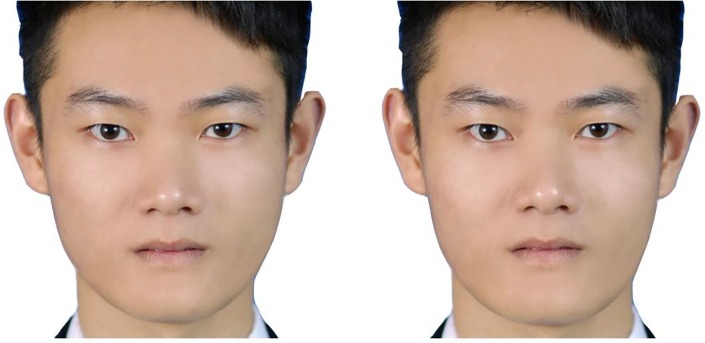
Examples of feminized (left) and masculinized (right) versions of a male face image used in this study. The written informed consent was obtained from the individual for the publication of this image.

Ten pairs of male faces that were created in a previous study (Zheng et al., 2013) were used to measure the facial masculinity preference. Twenty young adult Chinese male faces and 20 young adult Chinese female faces were used to create a male prototype face and a female prototype face, respectively. The 10 Chinese male face images were transformed by +50% of the differences in shape between the male and female prototypes to create masculinized versions and by −50% of the differences in shape to create feminized versions. Each pair of faces consisted of one version of a masculinized face and one version of a feminized face morphed from the same male face. The example stimuli are shown in Figure 1. Participants were asked to select which face was more attractive in each pair of faces. The proportion of masculinized faces chosen was calculated as the dependent variable.

#### Mental Rotation Ability

A short six-item three-dimensional mental rotation test, which was designed in a previous study (Zheng and Zheng, 2017), was used to measure the participants’ mental rotation ability. Items were selected from a mental rotation stimulus library (Peters and Battista, 2008). All diagrams were shown in white on a black background. Participants viewed the target diagram displayed on the top row and were asked to select the two matching comparison figures from four diagrams displayed on the bottom row. Following the procedure of previous studies (Zheng and Zheng, 2017), participants were asked to complete the task within 240 s, which included 30 s to complete each item, 30 s to read the introduction, and 30 s for the webpage to load. Participants could use the time freely and a countdown timer showed the amount of time that remained. The performance score was calculated by awarding a single point for each correct answer. Thus, the total score ranged from 0 to 12.

## Results

I conducted analyses of variance to examine group differences (heterosexual men, heterosexual women, gay men, and bisexual men) in the mental rotation ability and facial masculinity preference. There were significant differences in the mental rotation ability among groups, *F*(3, 809) = 17.9, *p* < 0.001, partial *η*^2^ = 0.062. Bonferroni *post hoc* test revealed that heterosexual women (*M* = 7.94, SD = 2.14) scored lower on mental rotation task than heterosexual men (*M* = 8.80, SD = 2.23, *d* = 0.4, *p* < 0.001); bisexual men (*M* = 9.22, SD = 1.73, *d* = 0.63, *p* < 0.001); and gay men (*M* = 9.09, SD = 1.94, *d* = 0.57, *p* < 0.001). There were no significant differences among other groups. There were also significant differences in facial masculinity preference among groups, *F*(3, 809) = 7.72, *p* < 0.001, partial *η*^2^ = 0.028. Bonferroni *post hoc* test revealed that gay men (*M* = 0.518, SD = 0.238) and bisexual men (*M* = 0.507, SD = 0.211) preferred more masculinized faces than heterosexual women (*M* = 0.427, SD = 0.222, *d* = 0.39/0.37, *p* < 0.001, *d* = 0.37, *p* = 0.017).

The association between the mental rotation ability and facial masculinity preference was significant for both the gay (*r* = 0.11, *p* = 0.045) and bisexual men (*r* = 0.22, *p* = 0.026) controlling for age, education, and occupation. There were no significant correlations between the mental rotation ability and facial masculinity preference in both heterosexual men (*r* = −0.054, *p* = 0.542) and women (*r* = 0.061, *p* = 0.342).

## Discussion

This study examined the association between the mental rotation ability and facial masculinity preference of gay and bisexual men. The results revealed that there was a significant association between mental rotation ability and facial masculinity preference in both gay and bisexual men, but not in heterosexual men or women.

There were significant gender differences in mental rotation ability, which is consistent with previous findings in western cultures (e.g., Voyer et al., 1995; Peters et al., 2007). However, there was no significant difference in mental rotation ability between heterosexual and homosexual men. Previous research has shown mixed findings with regard to sexual orientation differences in mental rotation ability. Some studies have found that heterosexual men outperform homosexual men in mental rotation ability (e.g., Rahman and Wilson, 2003; Maylor et al., 2007). Other research has shown no significant differences in mental rotation ability between heterosexual and homosexual men (e.g., Gladue et al., 1990). As for the facial masculinity preference, heterosexual women typically preferred more feminized male faces than did homosexual men. One recent study also indicated that women prefer feminized male faces in China (Liu and Wu, 2016).

The current findings indicated the association between mental rotation ability and facial masculinity preference. This association may be attributed to homogamy in partner preference in terms of masculinity in gay and bisexual men. Previous studies indicated that masculine gay and bisexual men tend to prefer masculine partners (Bailey et al., 1997; Zheng et al., 2013; Zheng and Zheng, 2015). Mental rotation ability is a masculine cognitive ability. Therefore, gay and bisexual men with high mental rotation ability would self-perceive more masculinity and prefer more masculine male faces. The current findings extend previous findings and indicate an association between masculine cognitive ability and facial masculinity preference, which was consistent with previous findings in heterosexual men that masculine cognitive style (i.e., systemizing) was related to facial masculinity preference (Smith et al., 2010). However, mental rotation was not associated with facial masculinity preference in heterosexual women. One previous study revealed no homogamy in facial masculinity-femininity in heterosexual couples (Burriss et al., 2011), which highlighted the homogamy theory in partner preference among gay and bisexual men.

If face processing ability could explain the association between mental rotation ability and facial masculinity preference, these associations should occur in both gay/bisexual men and heterosexual women. There was no significant association between mental rotation ability and facial masculinity preference in heterosexual women, which was consistent with a previous finding (Scarbrough and Johnston, 2005). This indicated that the association between mental rotation ability and facial masculinity preference could not be explained by face processing ability.

If gay and bisexual men’s preference for facial masculinity was based on intrasexual competition, mental rotation ability would be associated with facial masculinity preference in both gay/bisexual men and heterosexual men. There was no significant association between mental rotation ability and facial masculinity preference in heterosexual men, which was consistent with a previous finding (Vonnahme, unpublished). This indicated that preference for facial masculinity in gay and bisexual men was not based on intrasexual competition.

Mental rotation task performance is correlated with prenatal testosterone levels (Grimshaw et al., 1995), and relates to the ratio of the lengths of the second and fourth digits of the hand (a marker of prenatal exposure to androgens), which may reflect fetal exposure to prenatal sex hormones in early gestation (Burton et al., 2005; Collaer et al., 2007; Peters et al., 2007). Previous studies revealed the potential influences of prenatal androgen exposure on preference for exaggerated sex-typical characteristics in heterosexual men and women (Vonnahme, unpublished; Scarbrough and Johnston, 2005; Kuna and Galbarczyk, 2018). However, some recent studies did not find an association between hormone levels and facial masculinity preference (e.g., Jones et al., 2018a,b). The current findings may indicate the potential effects of prenatal androgen exposure on facial masculinity preference in gay and bisexual men.

There were several limitations in this study. First, the mental rotation task was conducted on a website rather than in a controlled laboratory environment. The devices that the participants used to complete the task may have influenced their performance because the display of the stimuli would be slightly different on a smartphone screen and desktop monitor. Second, as the current sample was young, the conclusion of this study is restricted to young gay and bisexual men in China. Third, the association between mental rotation ability and facial masculinity in gay and bisexual men was weak. The association between masculine cognitive ability and facial masculinity preference in gay and bisexual men should be tested with other abilities. Finally, the gay/bisexual participants and heterosexual participants were not recruited at the same time. This could have led to history effects.

## Data Availability Statement

All datasets generated for this study are included in the article/supplementary material.

## Ethics Statement

The studies involving human participants were reviewed and approved by the Academic Ethical Review Committee of Faculty of Psychology in Southwest University. Written informed consent from the participants was not required to participate in this study in accordance with the national legislation and the institutional requirements.

## Author Contributions

The author confirms being the sole contributor of this work and has approved it for publication.

### Conflict of Interest

The author declares that the research was conducted in the absence of any commercial or financial relationships that could be construed as a potential conflict of interest.

## Footnotes

^1^www.sojump.com
